# Volume creates value: The volume–outcome relationship in Scandinavian obesity surgery

**DOI:** 10.1177/09514848211048598

**Published:** 2022-02-06

**Authors:** Anna Svarts, Thorell Anders, Mats Engwall

**Affiliations:** 1 225274 Department of Industrial Economics and Management, KTH Royal Institute of Technology, Stockholm, Sweden; 2 27106 Department of Clinical Sciences, Karolinska Institutet, Danderyd Hospital, Stockholm, Sweden; 3 Department of Surgery, Ersta Hospital, Stockholm, Sweden

**Keywords:** Surgical volume, patient outcome, obesity, bariatric surgery, multilevel modeling, quality registry

## Abstract

This study establishes the relationship between surgical volume and cost and quality outcomes, using patient-level clinical data from a national quality registry for bariatric surgery in Sweden. Data include patient characteristics with comorbidities, surgical and follow-up data for patients that underwent gastric bypass or gastric sleeve operations between 2007 and 2016 (52,703 patients in 51 hospitals). The relationships between surgical volume (annual number of bariatric procedures) and several patient-level outcomes were assessed using multilevel, mixed-effect regression models, controlling for patient characteristics and comorbidities. We found that hospitals with higher volumes had lower risk of intraoperative complications as well as complications within 30 days post-surgery (odds ratios per 100 procedures are 0.78 and 0.87, respectively, *p*<0.01). In addition, higher-volume hospitals had substantially shorter procedure time (17 min per 100 procedures, *p*<0.01) and length of stay (0.88 incidence-rate ratio per 100 procedures *p*<0.01). Our results support the claim that increased surgical volume significantly improves quality. Further, the results strongly suggest that increased volume leads to lower cost per surgery, by reducing cost drivers such as procedure time and length of stay.

## Introduction

Anyone who learned to play a musical instrument knows that “practice makes perfect.” This intuitive idea constitutes the basis for research addressing the relationship between the quantity of healthcare provided by a hospital, and its outcome. Since this volume–outcome relationship was first addressed by Luft et al.,^1^ scholars have discussed the potential benefits of increased patient volume, often by means of geographical regionalization policy directing patients with specific conditions to designated hospitals, or by implementation of minimum volume standards that reduce the number of hospitals that provide specific services.^2–5^ Many empirical studies of surgical procedures have found a positive relationship between volume and outcome,^6–8^ with a stronger association for more complicated, or advanced, procedures.^9^ This is confirmed in various systematic reviews [e.g., Ref. 10]. The discussion has mainly focused on surgery, but the relationship is assumed to be present also in other areas such as internal medicine.^11^ While mechanisms and organizational factors that affect the volume–outcome relationship remain poorly understood,^12^ one underlying mechanism is assumed to be the “learning curve,” that is, that learning follows from greater experience.^13^ This has been described in a healthcare context for individuals,^14^ for teams and organizations,^15^ and as an interplay between the individual’s experience and a particular organization.^16^ However, the causality that increased volume has a positive effect on outcome has been questioned.^17^ It has been claimed that the causation might be reverse, that is, that higher-quality hospitals attract more patient referrals.^18^ Nevertheless, a recent study of German hospitals confirm the causality of the volume–outcome relationship and conclude that volume is the driving factor.^19^

Previous investigations of the volume–outcome relationship tend to have blunt approaches to measuring outcome.^4^ Bound by limitations of available data, most empirical studies use mortality as the (only) outcome measure.^20–24^ While mortality, obviously, is an important measure, quality in healthcare is a much more complex phenomenon, compromising both a patient safety dimension and an efficacy dimension.^25^ Mortality only partially captures patient safety, while, for example, other types of complications are ignored. In addition, despite the fact that efficient resource utilization is a key challenge for all healthcare provisioning, previous research has seldom included cost measures in the analysis of the volume–outcome relationship.

Consequently, based on the assumption that volume has a positive effect on outcome, the purpose of this paper is to establish

a. the relationship between surgical volume and surgical quality, using more comprehensive quality indicators than previous studies and
b. the relationship between surgical volume and cost per surgery.

## Methods

### Background

This paper is based on Swedish data from the Scandinavian Obesity Surgery Registry (SOReg). Obesity surgery, also called bariatric surgery or weight-loss surgery, is used to treat patients who are morbidly obese. There are two main surgical methods used today: (1) gastric bypass, in which nutrients, through rerouting, bypass most of the stomach, duodenum, and the proximal part of the small intestine and (2) sleeve gastrectomy, which involves resection of a major part of the stomach. In Sweden, data from over 99% of these operations are reported to the national quality registry, SOReg. The registry contains data on patient characteristics; surgical methods used; and short- as well as long-term outcomes. It constitutes one of the most comprehensive databases of bariatric surgery in the world and is continuously audited, demonstrating less than two percent of incorrect data entries.^26^

For this study, we collected data from the SOReg database for all primary gastric bypass and sleeve gastrectomies performed from January 1st 2007 to December 31st 2016. The study was approved by the SOReg registry steering committee and by the Regional Ethical Review Board in Stockholm, Sweden (registration number 2018/695-31/3).

### Dependent variables

The outcome variables included four quality indicators and two cost indicators.

For quality, we differentiated between patient safety and efficacy^25^ and included indicators for both of these dimensions: First, patient safety was measured as rates of complications, which is considered to be the most reliable measure of outcome quality in bariatric surgery, preferable to mortality, which is too rare to be a reliable measure.^27^ Thus, we included all complications that required surgical intervention under general anesthesia or intensive care (complication grade > 3a in the Clavien–Dindo classification system^28^) at three points in time: (1) complications during surgery (intraoperative complications); (2) complications within 30 days after surgery as reported at the follow-up visit approximately six weeks after surgery (30-day complications); and (3) complications occurring between 30 days and one year after surgery as reported at the follow-up visit approximately one year after surgery (one-year complications). In addition, clinical efficacy was measured as total weight loss one year after surgery in relation to pre-surgery weight (%TWL).

For estimation of costs, we used two indicators: (1) operating time in minutes (time from first skin incision to completion of wound closure) as a proxy for resource utilization during surgery and (2) length of stay, from hospital admission to discharge when patients return to their homes (LOS, in days), to measure resource utilization during the post-surgery hospital stay. Since prolonged times might be due to complications, both these time measures have been used as quality indicators in previous research.^29^ The measures are key drivers of hospital costs; the operating room represents one of the hospitals most costly resources; each minute of procedure time has been estimated to cost $22 to $80.^30,31^ Furthermore, post-surgery stays utilize both physical resources (beds, technical equipment, meals, etc.) and staff resources, and the longer the stay in the hospital, the more the resources are consumed.

### Explanatory variables

Surgical volume, that is, the annual number of primary bariatric surgical procedures performed at the hospital where surgery was performed, constituted the explanatory variable. To facilitate the interpretation of regression coefficients, we scaled this variable by division with 100, that is, one unit represents an annual volume of one hundred operations.

Furthermore, age, sex, pre-surgery BMI, and comorbidities (sleep apnea, hypertension, diabetes, and dyslipidemia) were included as covariates to control for case mix. Additional covariates were type of surgical procedure (gastric bypass or sleeve gastrectomy) and year of surgery. Bariatric surgery has become more established in Sweden during the time period included in the analysis, implying improved surgical technique (e.g., supported by the medical research facilitated by the quality registry, where differences in procedures are evaluated) and increased average experience-level of surgeons. Hence, we generally expect a trend toward improved results over time in the data and need to control for this by including year of surgery as a control variable.

### Description of data

We identified a total of 52,703 patients in the registry over the years 2007–2016. This excluded re-surgeries and patients that had other surgical procedures performed at the same time as the bariatric surgery. For comparability, we also excluded the few surgical procedures other than gastric bypass and sleeve gastrectomy performed during the study period (*n*=690) and patients younger than 18 years (*n*=31). Extreme values in the data were then checked to identify errors due to the manual data entry procedures. Some errors were found, mainly in relation to the length of stay variable, which depends on correct registration of both admission and discharge dates. All registry entries where procedure time was over 4 hours (*n*=105) and where LOS was longer than two weeks (*n*=195) were checked and in most cases explanations for the extreme values were found, in the occurrence of intraoperative complications (for procedure time) or postoperative complications (for LOS). When there was no explanation for an extreme value, the value was omitted from the analysis (i.e., treated as a missing value).

Table 1 shows descriptive statistics for the data set. Among the patients, 76% were female, the average age was 41 years, and the average pre-surgery body mass index (BMI) was 42 kg/m^2^. Gastric bypass was by far the most common surgical procedure, performed in 90% of patients. 1283 patients suffered from intraoperative complications, corresponding to a complications rate of 2.43%. 1934 patients had postoperative complications within 30 days, corresponding to 3.75%. 1395 patients had complications between 30 days and one year post-surgery, corresponding to 2.93%. Average total body weight loss after one year was 31%, with a standard deviation of 8%. On average, an operation lasted for 68.9 min and patients were discharged after 1.9 days.

**Table 1.** Descriptive statistics for bariatric surgery in Sweden 2007–2016.

*Patient level (N=52,703)*								
	Frequency (%)	Mean	Std. dev.	25^th^ percentile	Median	75^th^ percentile	Min.	Max.
Age		40.76	11.08	32	41	49	18	74
Female	40,123 (76)							
Male	12,580 (24)							
Gastric bypass	47,492 (90)							
Gastric sleeve	5211 (10)							
Pre-surgery BMI		42.02	5.52	38.14	41.26	45.00	27.77	86.56
Sleep apnea	5014 (9.5)							
Hypertension	12851 (24)							
Diabetes	7107 (14)							
Dyslipidemia	4934 (9.4)							
Procedure time		68.85	33.46	46	61	83	10	413
Length of stay		1.93	2.66	1	2	2	0	186
Intraoperative complications	1283 (2.4)							
30-day complications	1934 (3.7)							
One-year complications	1395 (2.9)							
%TW loss		0.31	0.078				−0.078	0.60

Hospital level (*N*=51).					
	*N*	Mean	Std. dev.	Min.	Max.
Surgical volume					
2007	26	30.39	31.80	1	108
2008	34	76.18	81.48	2	287
2009	36	107.14	104.41	1	438
2010	41	164.59	197.22	8	894
2011	42	180.24	199.76	1	977
2012	42	165.19	183.69	1	1056
2013	45	156.07	164.40	12	923
2014	43	146.67	151.71	15	897
2015	43	134.05	128.14	4	689
2016	44	116.27	118.39	4	591

During the studied period, the number of Swedish hospitals performing bariatric surgery increased from 26 in 2007 to 44 in 2016. As a few hospitals also ceased to perform bariatric surgery during the time period, a total of 51 unique hospitals were included in the study. Furthermore, the average number of bariatric procedures performed during one calendar year (surgical volume) increased from 30 per hospital to 116 per hospital, with a range from one single surgery, to a maximum of 1056 per hospital and year (see Appendix for the distribution of total number of procedures by year and hospital).

### Missing values

We employed a complete case analysis. With the exception of three variables, discussed below, less than 0.2% of the values for each variable were missing. Data on 30-day complications was missing for 2.1% of patients, reflecting a small number of individuals that either did not show up for the six-week follow-up visit, or had a follow-up visit at a different hospital, which did not report to the registry. For one-year complications and weight loss, 9.7 and 12.5% of the values were missing, respectively, presumably mainly reflecting patients that did not show up for the one-year follow-up visit. It is reasonable to suspect a bias effect for the weight-loss variable (%TWL): patients that do not have a satisfactory weight loss may not want to return to be weighed. However, the extent of missing values was deemed to be sufficiently low not to affect results.^32^ For one-year complications, there is less risk of a bias effect. Patients with serious complications could be assumed to return to the hospital; consequently, the patients with missing value for one-year complications are likely to belong to the great majority of patients without complications. A very small number of patients with missing one-year values were those who have died post-surgery. However, mortality is very low in the data set and this group represented only 27, out of the 5129 individuals with no one-year complications data.

### Statistical analyses

Multilevel regression analyses using complete case analysis were conducted using *STATA* version 14.1, with individuals nested within hospital-level random effects. Adopting a multilevel model structure has become increasingly common when comparing the performance of multiple healthcare providers.^33,34^ The model is based on the assumption that there are provider-specific “random” effects, which are drawn from a common distribution. In our case, the predictor variable was a hospital-level variable, while the output variables were measured at individual level, and this structure necessitated a multilevel modeling approach.^35^

Our multilevel models included random intercepts that allow different performance baselines for each hospital, as well as random slopes for the two variables “surgical volume” and “year,” which allows the effect of these variables to vary across hospitals. To validate the model specification, we used likelihood ratio tests, which showed improved model fit by adding random slopes for surgical volume and year (*p*<0.0001). Our data set did not allow for further random slopes without a break-down of the numerical estimation.

Logistic regression was used to assess complications, as it is a binary outcome. Linear regression was used to assess continuous data, that is, total weight loss (%TWL) and procedure time. Poisson regression was used to assess length of stay (LOS), as LOS is count values. All regressions used maximum likelihood (ML) estimation method. For the logistic and Poisson regression models, Stata’s standard mixed-effect ML estimation failed, and therefore, these models were estimated with Stata’s alternative estimation method using the QR decomposition of the variance-components matrix.

The relationships between surgical volume and outcome variables were inspected for non-linearity using scatterplots and locally weighted scatterplot smoothing curves of the pooled data. Since the plots showed a slight curve, which could be interpreted as either a linear relationship or a quadratic (U-shaped) relationship, we tried the inclusion of a quadratic term, that is, the square of surgical volume, in all model specifications. For intraoperative complications, six-week complications, procedure time, and length of stay the coefficients of the quadratic terms were very small and with low statistical significance (*p*-values 0.984, 0.048, 0.745, and 0.169), and these terms were therefore excluded. However, for one-year complications and percentage loss of total weight, the coefficients of the squared volume variable were significant (*p*-values 0.007 and 0.008) as well as larger and the quadratic terms were therefore included in the regression models.

## Results

The analysis shows that there is a statistically significant association between volume and all three patient safety quality outcomes (Table 2, part a). Volume has a significant positive effect on both intraoperative complications and postoperative complications within 30 days, with odds ratios of 0.78 and 0.87, respectively. This means that an increase by 100 procedures annually at a hospital corresponds to 22% lower risk of intraoperative complications as well as 13% lower risk of 30-day complications. The use of multivariate regression means that these odds ratios, as presented in Table 2, are adjusted for, for example, age, sex, BMI, and comorbidities. Volume also has a significant effect on complications that occur between 30 days and one year post-surgery, but this effect is non-linear and we find a lower risk of complications with increasing volume for low-volume hospitals, but a higher risk of complications with increasing volume for high-volume hospitals. Holding other factors constant, the turning point can be calculated to 452 procedures per year. (The turning point for the combined effect of β_1_X+β_2_X^2^ is the volume (x) where, holding other factors constant, the slope β_1_+2β_2_x = 0, that is, x = −β_1_/(2β_2_). The coefficients and natural logarithms of the odds ratios presented in Table 2a gives −(−0.200)/(2*0.0221)*100≈452).

**Table 2.** Association between surgical volume and outcome.

(a) Patient safety outcome									
	Intraoperative complications			30-day complications			One-year complications		
	Odds ratio^c^	95% CI	*p*-value	Odds ratio^c^	95% CI	*p*-value	Odds ratio^c^	95% CI	*p*-value
Surgical volume^a^	0.775^***^	0.690, 0.871	0.000	0.872^***^	0.806, 0.943	0.001	0.819***	0.707, 0.949	0.008
Surgical volume * surgical volume							1.022***	1.006, 1.039	0.007
Surgery characteristics									
Year	0.967	0.920, 1.016	0.181	0.891^***^	0.853, 0.930	0.000	0.974	0.920, 1.032	0.374
Gastric bypass^b^	1.822^***^	1.372, 2.419	0.000	1.346^**^	1.057, 1.714	0.016	3.599***	2.518, 5.142	0.000
Patient characteristics									
Age	1.019^***^	1.013, 1.025	0.000	1.013^***^	1.008, 1.017	0.000	0.983***	0.978, 0.989	0.000
Female	0.898	0.787, 1.024	0.108	0.948	0.849, 1.059	0.347	1.045	0.911, 1.198	0.532
Pre-surgery BMI	1.018^***^	1.007, 1.029	0.001	0.992^*^	0.983, 1.001	0.092	0.963***	0.952, 0.974	0.000
Comorbidities									
Sleep apnea	0.879	0.721, 1.073	0.206	1.079	0.921, 1.263	0.347	1.092	0.891, 1.339	0.396
Hypertension	1.139^*^	0.983, 1.319	0.083	0.958	0.846, 1.085	0.501	0.894	0.762, 1.049	0.169
Diabetes	1.119	0.946, 1.324	0.191	1.054	0.914, 1.215	0.473	0.837*	0.690, 1.016	0.072
Dyslipidemia	0.930	0.762, 1.134	0.473	0.858^*^	0.721, 1.021	0.085	0.861	0.680, 1.091	0.216
Random effects									
Surgical volume (std. deviation)	0.152^***^	0.0870, 0.266	0.000	0.0658^***^	0.0172, 0.252	0.000	0.149***	0.0828, 0.269	0.000
Surgical volume^b^ * surgical volume^b^ (std. deviation)							5.33 × 10^−10^		
Year (std. deviation)	0.131^***^	0.0917, 0.188	0.000	0.113^***^	0.0803, 0.160	0.000	0.162***	0.121, 0.217	0.000
Constant (std. deviation)	0.384^***^	0.266, 0.553	0.000	0.453^***^	0.341, 0.603	0.000	0.0726	0.00052, 10.1	0.297
Model									
Intraclass correlation (ICC)	0.0428			0.0588			0.00160		
Observations (*N*)	52 703			51 575			47 574		

(b) Clinical efficacy outcome			
	% total weight loss after one year		
	Coefficient	95% CI^d^	*p*-value
Surgical volume^a^	0.00435^***^	0.00220, 0.00651	0.000
Surgical volume * surgical volume	−0.000253^***^	−0.000439, −0.0000668	0.008
Surgery characteristics			
Year	0.00235^***^	0.00143, 0.00327	0.000
Gastric bypass^b^	0.0569^***^	0.0473, 0.0664	0.000
Patient characteristics			
Age	−0.00127^***^	−0.00139, −0.00115	0.000
Female	0.0190^***^	0.0174, 0.0207	0.000
Pre-surgery BMI	0.00164^***^	0.00144, 0.00183	0.000
Comorbidities			
Sleep apnea	−0.00325^**^	−0.00616, −0.000337	0.029
Hypertension	−0.00140	−0.00359, 0.000793	0.211
Diabetes	−0.0272^***^	−0.0299, −0.0244	0.000
Dyslipidemia	−0.00469^***^	−0.00699, −0.00239	0.000
Constant	0.230^***^	0.217, 0.244	0.000
Random effects			
Surgical volume (std. deviation)	2.06 × 10^−8^		
Surgical volume * surgical volume (std. deviation)	1.47 × 10^−9^		
Year (std. deviation)	0.00264^***^	0.00150, 0.00464	0.000
Constant (std. deviation)	0.0106^***^	0.00763, 0.0147	0.000
Residual (std. deviation)	0.0711^***^	0.0699, 0.0724	0.000
Model			
Intraclass correlation (ICC)	0.0218		
Observations (*N*)	46 113		

(c) Cost driver outcome						
	Procedure time			Length of stay		
	Marginal effect	95% CI^d^	p-value	Incidence-rate ratio^c^	95% CI	p-value
Surgical volume^a^	−17.41***	−23.17, −11.65	0.000	0.881***	0.820, 0.946	0.001
Surgery characteristics						
Year	−6.213***	−7.575, −4.851	0.000	0.904***	0.889, 0.920	0.000
Gastric bypass^b^	10.49***	5.441, 15.55	0.000	0.838***	0.814, 0.863	0.000
Patient characteristics						
Age	0.100***	0.0744, 0.126	0.000	1.003***	1.002, 1.004	0.000
Female	−5.441***	−6.443, −4.438	0.000	1.033***	1.018, 1.049	0.000
Pre-surgery BMI	0.381***	0.282, 0.480	0.000	1.004***	1.003, 1.006	0.000
Comorbidities						
Sleep apnea	0.465	−0.646, 1.577	0.412	1.060***	1.037, 1.084	0.000
Hypertension	0.875***	0.337, 1.413	0.001	0.987	0.970, 1.004	0.121
Diabetes	2.418***	1.624, 3.212	0.000	1.088***	1.066, 1.109	0.000
Dyslipidemia	0.119	−0.563, 0.800	0.733	1.011	0.988, 1.035	0.340
Constant	79.66***	72.14, 87.18	0.000	2.018***	1.786, 2.281	0.000
Random effects						
Surgical volume (std. deviation)	18.15***	13.63, 24.18	0.000	0.219***	0.166, 0.289	0.000
Year (std. deviation)	4.668***	3.448, 6.320	0.000	0.0565***	0.0448, 0.0712	0.000
Constant (std. deviation)	26.99***	22.31, 32.64	0.000	0.354***	0.285, 0.439	0.000
Residual (std. deviation)	21.90***	19.25, 24.91	0.000			
Model						
Intraclass correlation (ICC)	0.603					
Observations (*N*)	52 687			52 618		

95% confidence intervals. ^*^ *p* < 0.1, ^**^ *p* < 0.05, ^***^ *p* < 0.01.

^a^ Surgical volume in 100s surgeries per year.

^b^ Surgical procedure was either gastric bypass or sleeve gastrectomy.

^c^ Exponentiated coefficients.

^d^ Calculated using robust standard errors.

Table 2, part (b), shows that there is a statistically significant positive association between volume and clinical efficacy measured as percentage loss of total weight (%TWL). Although statistically significant, the volume effect on weight loss is very small. There is also a statistically significant, although even smaller, negative effect of the squared volume on %TW, which signifies a non-linear relationship between volume and efficacy: the relationship weakens at higher volumes, until it reaches an optimum where additional volume will have a negative effect. The linear coefficient of 0.44 percentage and the quadratic coefficient of −0.025 percentage implies that volume will, theoretically, have a positive effect on weight loss up to an annual volume of approximately 860 procedures. (The turning point x = −β_1_/(2β_2_) is −0.00,435/(2*–0.000,253)*100≈860). This turning point is at the extreme end of the volume range in our data set, where only a few annual volumes at one of the hospitals exceed 860 procedures. This indicates an overall positive relationship, within the volume range that we have studied, but with a weakening effect at higher volumes.

Finally, part (c) of Table 2 shows that there is a statistically significant association between volume and costs. The marginal effect on procedure time is estimated to −17.4 min, which is a significant value, compared to the reported mean procedure time of 68.9 min. In addition, the incidence-rate ratio for 100 procedures is 0.88 for length of stay (LOS), which corresponds to a decrease of 12%, that is, that the average number of days a patient has to stay in ward of the hospital decreases from 1.93 days to 1.70 days.

Although we were mainly interested in the population-level effect of surgical volume, the random effects shown in Table 2 suggest that the volume effect varies between hospitals. In particular, for the cost drivers, there are large differences: the standard deviation of the effect of surgical volume on procedure time between different hospitals was 18.2 min. When compared to the overall effect of 17.4 min, this implies that the volume effect was very strong in some hospitals, but negligible in others.

The intraclass correlation coefficients (ICCs) varied from 4 to 6% for complications and 2% for %TWL, to 60% for procedure time. Since only around 5% of the variation was explained by hospital-level factors, this means that most of the variance of quality outcomes depended on individual patient factors, However, hospital-level factors were more important than individual patient characteristics for explaining variation in procedure time.

## Discussion

This study addresses the volume–outcome relationship in surgery. Based on more comprehensive quality indicators than previous studies, the purpose is to investigate the relationship between (a) surgical volume and quality as well as (b) surgical volume and cost per surgery. Our findings confirm previous research: increased surgical volume has a positive effect on quality.

First, there is a significant volume effect on patient safety for complications that occur during and after the operation. However, for complications reported one year after the surgery, we found that volume was only associated with reduced complications up to an annual volume of around 450 procedures. The positive volume effect at lower volumes is in line with our overall findings and previous research. We have no clear explanation for the negative volume effect at higher volumes. It might be speculated that patients with more severe comorbidity are more likely to be referred to hospitals with high volumes, which in turn increases the risk of late complications, since complications after the first few months often are related to medical conditions associated with patient’s comorbidities and life style, rather than caused by the surgical procedure per se. Furthermore, very few hospitals in our data set had annual volumes of more than 450 procedures (see Supplementary File A1 in Appendix) and this may affect the result.

Second, there is a significant volume effect on efficacy, measured as weight loss (%TWL). However, since the 0.44 percentage point coefficient indicates a very small effect, this statistical significance is probably not clinically relevant. As an example, for a patient with a preoperative weight of 140 kg, the standard weight loss of 30% after one year corresponds to a new weight of 98 kg. If this patient had a surgery at a hospital with 200 more procedures, the new weight would be 97.1 kg. This difference of 0.9 kg is not clinically meaningful.^36^ Furthermore, the non-linear relationship means that this is the maximum effect size; the effect is even smaller for hospitals with larger volumes.

Moreover, and importantly, our findings show that surgical volume has a strong positive effect on the two cost drivers (1) procedure time in the operating theater and (2) patient length of postoperative stay (LOS). This clearly suggests that higher-volume hospitals have lower costs per surgery. However, the effect on procedure time is highly variable between different hospitals, indicating that the individual learning curves varies between organizations. These differences may be explained by contextual factors outside the scope of the study, such as differences in staff turnover, educational level, and working processes. However, these findings indicate a need for future research that further our understanding of procedure time and its relationship to quality and cost. The quality registry used in the present study contains data on both procedure time and long-term and short-term complications; hence, this type of registry would provide a good empirical basis for such analysis.

In addition, the variable relationship between volume and procedure time also indicates a need for further research on factors affecting learning in healthcare organizations. For example, Sakai-Bizmark et al.^4^ found that higher-volume hospitals in the US had increased LOS after pediatric cardiac surgeries and proposed this to be an effect of regionalization of high-volume hospitals, that is, that patients from more distant regions stay longer at the hospital. Our findings, however, given modest travel distances in Swedish healthcare, suggest that reduced frequency of complications and well-designed after-care processes allow higher-volume hospital departments to reduce costs by shorter length of stay.

The present study shows that hospitals with higher volumes of bariatric surgical procedures have better outcomes. There are two possible explanations of this relationship: volume might drive performance, or performance might drive volume. In the former case, volume enables individual and organizational learning resulting in improved procedures, which result in better outcomes. In the latter case, good outcome records result in stronger demand or more referrals, which result in higher volumes at the hospitals with good outcomes. The causal direction might be due to the context of the healthcare operations. In Sweden, the individual choice of healthcare provider is limited, and the majority of obesity surgery patients whose procedures are tax-funded are typically remitted to a closely located provider. Given this empirical context, it is plausible to assume that volume drives performance in this setting rather than the reversed causality. This is in line with the recent study by Hentschker and Mennicken (19) who use causal methods to establish the causation from volumes to outcomes in the context of German hospital care. However, these dynamics might differ between different contexts and healthcare systems; hence, these contextual factors ought to be taken into account in further research.

A key strength of the present study is its access to a large cohort of patients registered in a database with a comprehensive set of relevant variables with high follow-up rates. However, the study has some limitations. First, it uses data from the Scandinavian Obesity Surgery Registry, and this may limit generalizability to other geographic regions. This might be particularly true for developing countries, where conditions for quality and cost in healthcare are substantially different from those in Scandinavia. It is plausible that our results are valid for countries in North America and Europe with similar conditions as in Sweden, but this needs to be confirmed by future research. Second, transferability of our results to other surgical settings might also be limited. Since bariatric surgery is a complicated type of surgery, the volume effect for procedures that are less complex, and associated with lower risk of complications, might be less significant.^37^ Furthermore, a third limitation is that our study investigates the effect of volume without differentiating between hospitals where bariatric surgery is a small subset of all surgical procedures, and hospitals where bariatric surgery represents a major part of the total volume. Specialty hospitals with a narrow scope, primarily focusing on bariatric surgery, probably experience a faster learning curve effect than general hospitals with a broad scope, covering a wide spectrum of various surgical interventions.^38^

The present findings have implications for the ongoing debate on regionalization of healthcare. Our results suggest that regionalizing as a means to increase individual hospitals’ surgical volumes can increase patient safety, and maybe also efficacy, while lowering unit costs. This relates to two of the three goals in the “iron triangle” of healthcare delivery: access, quality, and cost.^39^ However, previous research has also shown that regionalization can hamper patient access to care, for instance, for patients in rural areas, with low mobility, or belonging to weak socio-economic groups.^2,40^ Thus, there is a tradeoff between healthcare access, on the one hand, and quality and cost, on the other. Currently, socio-economic factors do not affect the likelihood of receiving bariatric surgery in Sweden.^41^ However, the risk of affecting the equal access to healthcare needs to be acknowledged when concentration of healthcare services, for example, bariatric surgery, to a smaller number of hospitals is considered.

Furthermore, it is important to distinguish between surgical volume and hospital size in discussions of geographical regionalization. Our findings demonstrate the benefits of high surgical volumes. However, high surgical volumes could be found in large, general hospitals, as well as in small surgical centers specialized to achieve high volumes for one specific type of surgical procedure. Thus, to guide policy makers, further studies are needed, where surgical volume, specialization, and hospital size as predictors of outcome are included.

## Conclusion

This study shows that in bariatric surgery, higher surgical volume is associated with improved quality in terms of patient safety and efficacy, as well as with reduced costs per procedure. While the volume effect on patient safety has been established previously, the present findings reveal the volume effect on efficacy and costs. However, it is important to distinguish between surgical volume and hospital size. Since small hospitals can specialize to achieve high volumes in specific surgical procedures, higher surgical volume does not necessarily mean larger hospitals. Nonetheless, the results imply that a concentration of surgical volumes to a few, well focused healthcare units—irrespective if these departments belong to large general hospitals or small specialty hospitals—leads to improved quality and lower cost per surgery.

## Supplemental Material

sj-pdf-1-hsm-10.1177_09514848211048598 – Supplemental Material for Volume creates value: The volume–outcome relationship in Scandinavian obesity surgery Click here for additional data file. Supplemental Material, sj-pdf-1-hsm-10.1177_09514848211048598 for Volume creates value: The volume–outcome relationship in Scandinavian obesity surgery by Anna Svarts, Thorell Anders and Mats Engwall in Health Services Management Research

## Figures and Tables

**Figure A.1:**
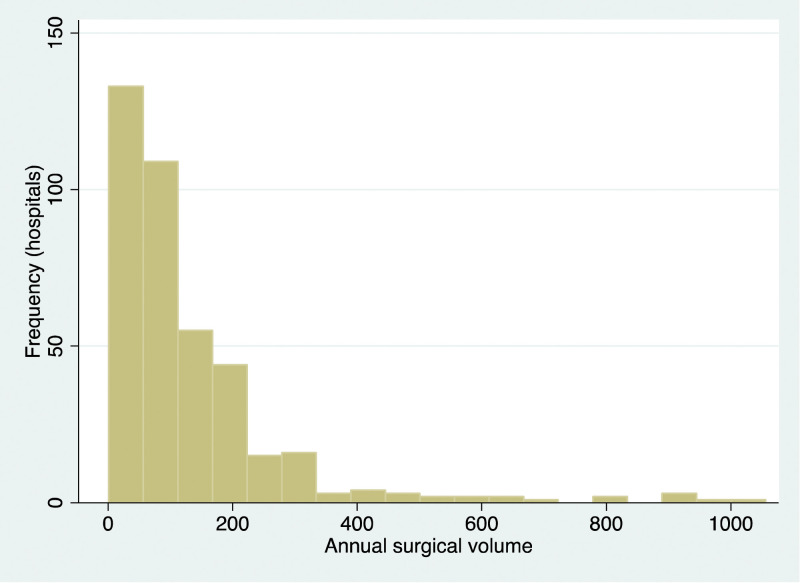
Distribution of annual surgical volume (count of hospitals). Figure A.1 shows the number of hospitals with different surgical volumes (hospitals that were operational throughout the time period 2007-2016 are counted ten times in the histogram).

**Figure A.2:**
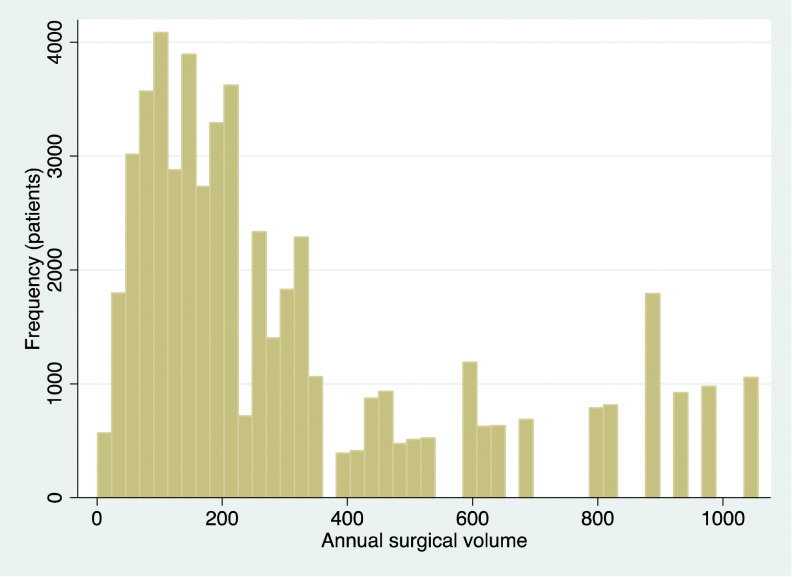
Distribution of annual surgical volume (count of patients). Figure A.2 shows the number of patients that underwent bariatric surgery in hospitals with different surgical volumes. The volume reported in the histogram is the annual volume of the year when the patient underwent surgery.
