# Supplementary material for: Volume creates value: The volume–outcome relationship in Scandinavian obesity surgery
Source: Health Serv Manage Res. 2022 Feb 6;35(4):229–39. doi: 10.1177/09514848211048598 (PMC9574905; doi:10.1177/09514848211048598)
Supplement: sj-pdf-1-hsm-10.1177_09514848211048598 – Supplemental Material for Volume creates value: The volume–outcome relationship in Scandinavian obesity surgery [file sj-pdf-1-hsm-10.1177_09514848211048598.pdf]

Appendix

Figure A.1: Distribution of annual surgical volume (count of hospitals)

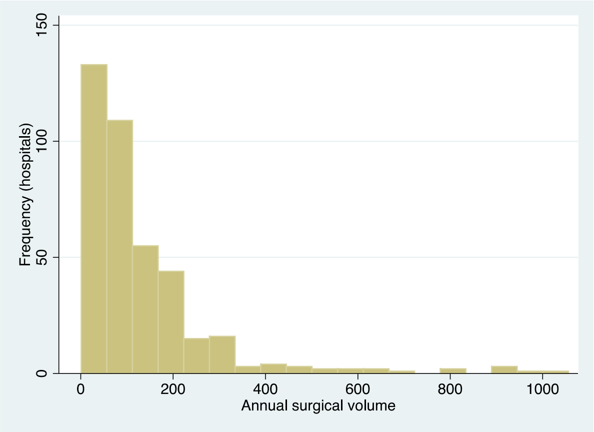

Figure A.1 shows the number of hospitals with different annual surgical volumes (hospitals that were operational throughout the time period 2007-2016 are counted ten times in the histogram).

Figure A.2: Distribution of annual surgical volume (count of patients)

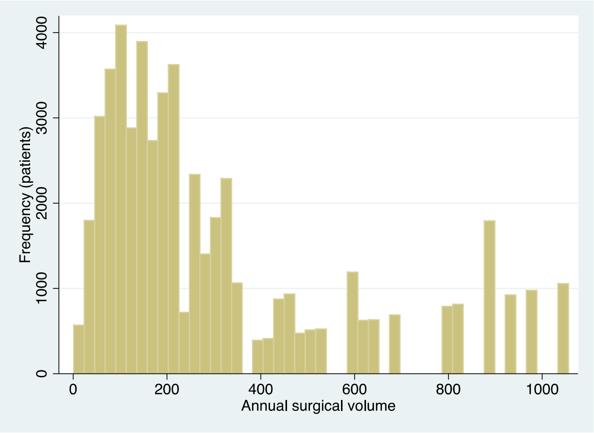

Figure A.2 shows the number of patients that underwent bariatric surgery in hospitals with different annual surgical volumes. The volume reported in the histogram is the annual volume of the year when the patient underwent surgery.
